# Kallikrein 5 Inhibition by the Lympho-Epithelial Kazal-Type Related Inhibitor Hinders Matriptase-Dependent Carcinogenesis

**DOI:** 10.3390/cancers13174395

**Published:** 2021-08-31

**Authors:** Elaine Zayas Marcelino da Silva, Thais Fernanda de Campos Fraga-Silva, Yao Yuan, Márcia Gaião Alves, Gabriel Azevedo Publio, Carol Kobori da Fonseca, Márcio Hideki Kodama, Gabriel Viliod Vieira, Marina Ferreira Candido, Lara Maria Alencar Ramos Innocentini, Mateus Gonçalves Miranda, Alfredo Ribeiro da Silva, Jose Carlos Alves-Filho, Vania Luiza Deperon Bonato, Ramiro Iglesias-Bartolome, Katiuchia Uzzun Sales

**Affiliations:** 1 Department of Cell and Molecular Biology and Pathogenic Bioagents, Ribeirao Preto Medical School, University of São Paulo, Ribeirao Preto 14049-900, SP, Brazil; ezmdasilva@alumni.usp.br (E.Z.M.d.S.); alvesg.marcia@gmail.com (M.G.A.); kobori@usp.br (C.K.d.F.); marciohkodama@gmail.com (M.H.K.); gabrielviliod@gmail.com (G.V.V.); mahfcandido@gmail.com (M.F.C.); mateusgm@usp.br (M.G.M.); 2 Basic and Applied Immunology Program, Department of Biochemistry and Immunology, Ribeirao Preto Medical School, University of São Paulo, Ribeirao Preto 14049-900, SP, Brazil; thaisfragasilva@usp.br (T.F.d.C.F.-S.); vlbonato@fmrp.usp.br (V.L.D.B.); 3 Laboratory of Cellular and Molecular Biology, Center for Cancer Research, National Cancer Institute, National Institutes of Health, Bethesda, MD 20892, USA; yao.yuan2@nih.gov (Y.Y.); ramiro.iglesias-bartolome@nih.gov (R.I.-B.); 4 Departament of Pharmacology, Ribeirao Preto Medical School, University of São Paulo, Ribeirao Preto 14049-900, SP, Brazil; gabriel.publio@usp.br (G.A.P.); jcafilho@usp.br (J.C.A.-F.); 5 Dentistry and Stomatology Division, Ophthalmology, Otolaryngology, and Head and Neck Surgery Department, Clinical Hospital of Ribeirao Preto Medical School, University of São Paulo, Ribeirao Preto 14049-900, SP, Brazil; lara.m.alencar@hotmail.com; 6 Department of Pathology and Legal Medicine, Ribeirao Preto Medical School, University of São Paulo, Ribeirao Preto 14049-900, SP, Brazil; arsilva@fmrp.usp.br

**Keywords:** LEKTI, SPINK5, KLK5, OSCC, matriptase

## Abstract

**Simple Summary:**

Head and neck squamous cell carcinomas (HNSCC) are among the most common cancers worldwide. In contrast to the advances in prevention and treatment of other types of cancer, the five-year survival rate for HNSCC is only about 50% and it has not changed for the past 50 years. This poor prognosis is mainly due to a shortage of suitable markers for early detection, delayed diagnosis and/or referral, and ineffectiveness of chemotherapy. The aim of this study was to explore the inhibitory role of LEKTI in matriptase-dependent squamous cell carcinogenesis and to investigate additional players operating in this pathway. We found that Kallikrein-5 is necessary for PAR-2-mediated IL-8 release, YAP1-TAZ/TEAD activation, and matriptase-mediated oral squamous cell carcinoma migration. This knowledge can contribute for the development of future targeted therapy in HNSCC.

**Abstract:**

Head and neck squamous cell carcinoma remains challenging to treat with no improvement in survival rates over the past 50 years. Thus, there is an urgent need to discover more reliable therapeutic targets and biomarkers for HNSCC. Matriptase, a type-II transmembrane serine protease, induces malignant transformation in epithelial stem cells through proteolytic activation of pro-HGF and PAR-2, triggering PI3K-AKT-mTOR and NFKB signaling. The serine protease inhibitor lympho-epithelial Kazal-type-related inhibitor (LEKTI) inhibits the matriptase-driven proteolytic pathway, directly blocking kallikreins in epithelial differentiation. Hence, we hypothesized LEKTI could inhibit matriptase-dependent squamous cell carcinogenesis, thus implicating kallikreins in this process. Double-transgenic mice with simultaneous expression of matriptase and LEKTI under the keratin-5 promoter showed a prominent rescue of K5-Matriptase^+/0^ premalignant phenotype. Notably, in DMBA-induced SCC, heterotopic co-expression of LEKTI and matriptase delayed matriptase-driven tumor incidence and progression. Co-expression of LEKTI reverted altered Kallikrein-5 expression observed in the skin of K5-Matriptase^+/0^ mice, indicating that matriptase-dependent proteolytic pathway inhibition by LEKTI occurs through kallikreins. Moreover, we showed that Kallikrein-5 is necessary for PAR-2-mediated IL-8 release, YAP1-TAZ/TEAD activation, and matriptase-mediated oral squamous cell carcinoma migration. Collectively, our data identify a third signaling pathway for matriptase-dependent carcinogenesis in vivo. These findings are critical for the identification of more reliable biomarkers and effective therapeutic targets in Head and Neck cancer.

## 1. Introduction

Head and neck squamous cell carcinomas (HNSCC) are among the most common cancers worldwide, with over 800,000 new cases diagnosed yearly [1]. In contrast to the advances in prevention and treatment of other types of cancer, the five-year survival rate for HNSCC is only about 50% and is unchanged for 50 years [2,3]. Generally, this poor prognosis is due to a shortage of suitable markers for early detection, delayed diagnosis and/or referral, and ineffectiveness of chemotherapy [3,4,5]. Accordingly, there is an urgent need to identify more reliable therapeutic targets and biomarkers of prognosis in HNSCC.

Several studies demonstrated that dysregulated expression of Type II Transmembrane Serine Proteases (TTSPIIs) is associated with different types of cancers [6]. Matriptase, a TTSPII, is indeed dysregulated in several cancers [7]. List et al. (2005) showed that Matriptase induces malignant transformation when expressed in epithelial stem cells [8]. It has been shown that PI3K-Akt-mTor signaling, elicited by proteolytic activation of pro-hepatocyte growth factor (pro-HGF), is a molecular pathway by which matriptase promotes malignant transformation [9]. Another essential component of matriptase-mediated oncogenesis is the upregulation of NFkB-induced inflammatory cytokines dependent on Protease Activated Receptor 2 (PAR-2) proteolytic cleavage by matriptase [10].

Matriptase was also implicated in Netherton Syndrome (NS), an epidermal disorder caused by mutations in the SPINK5 gene, which encodes for the Lympho-Epithelial Kazal-Type-related Inhibitor (LEKTI). LEKTI inhibits matriptase-dependent skin desquamation in NS by the direct inhibition of Kallikreins 5 (KLK5) and 7 (KLK7), which are known for their role in the degradation of corneodesmosomes at the outermost layers of the stratum corneum [11]. Kallikrein-related peptidases (KLKs) comprise a large family of secreted serine proteases expressed in several tissues [12,13]. Kallikreins are secreted as inactive zymogens and generally depend on proteolytic cleavage at the carboxy-terminal end of either arginine or lysine for activation [14] and are often dysregulated in inflammatory skin disorders and many human cancers [12,15]. Abnormal functional levels of the KLKs in diseased states are a result of unbalanced activation and inhibition events. These peptidases function cooperatively in signaling cascades or complex regulatory networks, often bridging multiple protease families and classes [16] Accordingly, matriptase is able to activate pro-KLK5 and pro-KLK7 [11].

In this study, we found a decrease in LEKTI expression in poorly differentiated OSCCs. This led us to the hypothesis that LEKTI could be a tumor suppressor by regulating KLK activation by matriptase. Indeed, double-transgenic mice with simultaneous expression of matriptase and LEKTI under the keratin-5 promoter led to the inhibition of matriptase-dependent carcinogenesis through downregulation of KLK5 expression. Significantly, KLK5 promoted the release of IL-8 and activated the Yap1-TAZ/TEAD transcription network through PAR-2 activation. In conclusion, our study identifies a novel proteolytic pathway that contributes to the matriptase-driven malignant transformation.

## 2. Material and Methods

### 2.1. Human Tissue Samples

In this study, 127 cases of human Oral Squamous Cell Carcinomas (OSCCs) diagnosed between 2005 and 2016 were selected from Pathology Service (SERPAT) of Ribeirao Preto Medical School, University of São Paulo, to compose a tissue microarray (TMA). Formalin-fixed and paraffin-embedded TMAs were composed of well-differentiated OSCCs, W.D.C., *n* = 37; moderately-differentiated OSCCs, M.D.C., *n* = 59; and poorly-differentiated OSCCs, P.D.C., *n* = 31. Only cases whose diagnoses were made in the following regions of the oral cavity were considered: jaw, floor of mouth, retromolar, mouth, oral cavity, tongue, cheek mucosa, gingiva, gum, larynx/pharynx, hard palate, and lips.

### 2.2. Tissue Microarray Construction

An experienced pathologist (ARS) reviewed the hematoxylin and eosin slides to delineate the most significant area of each tumor to prepare the TMA. From the preselected donor paraffin blocks, 2 mm diameter cylinders were removed and sorted into a paraffin block recipient using the TMA Builder Kit (Histopathology Ltd., Pécs, Hungary).

### 2.3. Ethics Statement

The study was performed in accordance with the Declaration of Helsinki [17] and approved by the Ethics Committee on Human Research of Ribeirao Preto Clinical Hospital and Ribeirao Preto Medical School, University of São Paulo (protocol: 50533515.6.0000.5440, approved on 21 June 2016). Histopathological analysis was performed on paraffin-embedded non-identified samples comprising incisional biopsies. In this situation, patient consent is waived according to Brazilian laws.

The K5-LEKTI mice were generated in accordance with protocols approved by the National Institute of Dental and Craniofacial Research Animal Care and Use Committee. All mice were kept and bred in the animal facility of the Department of Cell and Molecular Biology and Pathogenic Bioagents, Ribeirao Preto Medical School, University of São Paulo, Brazil. All experiments involving mice were approved by the Ethics Committee on Animal Research of Ribeirao Preto Medical School, University of São Paulo (protocol: 003/2015-1, approved on 29 June 2015) and were performed in accordance with the Guidelines of the Brazilian College of Animal Experimentation.

### 2.4. Mice

The generation of Keratin5-matriptase transgenic mice (K5-Matriptase^+/0^) has been described [8]. Keratin5-LEKTI transgenic mice (K5-LEKTI^+/0^) were generated by cloning the full-length 3-kb mouse LEKTI cDNA (NM_001081180.1) into the pBK5-vector containing the 5.2-kb bovine keratin-5 regulatory sequences, beta-globin intron-2, and 3′-polyadenylation sequences [18]. The linearized vectors were microinjected into the male pronucleus of FVB zygotes, which were implanted into pseudopregnant mice. Founder animals carrying the transgene were identified by PCR analysis of genomic DNA extracted from tail biopsies. WT and K5-Mariptase^+/0^/K5-LEKTI^+/0^ were generated by interbreeding of K5-Mariptase^+/0^ with K5-LEKTI^+/0^ mice. The study was strictly littermate controlled to avoid genetic background differences from confounding data interpretation. The transgenic mice were genotyped by PCR of genomic DNA extracted from tail biopsies as described previously [19].

### 2.5. Cells

The HNSCC cell line Cal 27 was a kind gift from Dr. J. Silvio Gutkind (University California San Diego, San Diego, CA, USA). Cal 27 are derived from a human tongue SCC [20]. Generation of Cal 27 KLK5 knockout cells was previously reported [21]. Both WT and KLK5 knockout Cal 27 cells were authenticated by STR profiling (DATAPEP-FMUSP, Sao Paulo, SP, Brazil) with the following results: TH01 6, 9.3; D5S818 11, 12; D13S317 10, 11; D7S820 10; D16S539 11, 12; CSF1PO 10, 12; vWA 14, 17; TPOX 8; Amelogenin X. HEK293T cells are from ATCC and were not further authenticated (Catalog# CRL-3216, Manassas, VA, USA). Cells were cultured as previously described [21].

### 2.6. Antibodies and Recombinant Proteins

The following primary antibodies were used for IHC: anti-SPINK5 (3 µg/mL; clone HPA009067, Merck, Darmstadt, Germany), anti-Matriptase-ST14 (1:400 dilution, Catalog# AF3946, R&D Systems, Minneapolis, MN, USA) and anti-mouse Kallikrein 5 (1:800 dilution; Catalog# MAB7236, R&D Systems). The following biotinylated secondary antibodies were used: goat anti-mouse IgG, goat anti-rat IgG, rabbit anti-sheep IgG (1:400; Vector Laboratories, Burlingame, CA, USA). The following conjugated primary antibodies were used for flow cytometry: anti-CD45-PeCy7 (clone 30-F11), anti-Ly6G-APC (clone IA8), anti-CD11c-FITC (clone HL3), anti-MHCII-BB700 (clone M5/114.15.2) from BD Bioscience (Franklin Lakes, NJ, USA), and anti-CD64-APC-Cy7 (clone X54-5/7.1) and anti-CD16/CD32 monoclonal antibody (clone 93) from eBioscience (San Diego, CA, USA). The following human recombinant proteases were used: Recombinant Human Matriptase/ST14 Catalytic Domain (Catalog# 3946-SE) and Recombinant Human Kallikrein 5 Protein (Catalog# 1108-SE), from R&D Systems.

### 2.7. SPINK5 mRNA Expression Analysis in K5-LEKTI^+/0^ Mouse Epidermis

Newborn WT and K5-LEKTI^+/0^ FVB/NJ mice were euthanized by decapitation. Dissected skin was incubated in PBS containing 10 mM EDTA for 5 min at 56 °C to separate the dermis from the epidermis. The epidermis was immediately covered with RNALater (MilliporeSigma, Burlington, MA, USA), and incubated overnight at 4 °C. For the extraction of total RNA, we used TRIzol (Thermo Fisher Scientific, Waltham, MA, USA). Epidermis was added to 1 mL of TRIzol buffer and 100 mg of Precellys^®^ zirconium oxide beads (Bertin Technologies, Montigny-le-Bretonneux, France). The samples were homogenized using a Precellys-24 tissue homogenizer (Bertin Technologies) for three pulses at 6800 rpm for lysis and homogenization. The samples were then transferred to a new RNase/DNase-free tube for chloroform extraction. The upper phase was transferred to a new tube for isopropanol precipitation. Samples were vortexed and centrifuged at 12,000× *g* for 30 min. The pellet was then washed with 70% ethanol in DEPC water, and the material was dried. RNA was resuspended in DEPC water. RNA samples were quantified, and cDNA synthesis was performed from 1 μg total RNA using the Kit ™cDNA Synthesis SuperScript^®^ VILO (Catalog# 11754, Thermo Fisher Scientific). The qRT-PCR reaction was performed in the 7500 Real-Time PCR System according to the protocol of TaqMan Master Mix (Applied Biosystems, Thermo Fisher Scientific) manufacturer. Gapdh and Hprt1 were used as internal controls. LEKTI gene expression was evaluated using mouse SPINK5 TaqMan probes (Mm00486343_cn). All reactions were repeated three times, and the experiments were validated with the use of negative controls.

### 2.8. Histological and Immunohistochemical Analysis

Adult mice were euthanized by CO_2_ inhalation, and newborn mice were euthanized by decapitation. Mouse tissues were fixed overnight in 4% paraformaldehyde and embedded in paraffin. Both mice and TMA paraffin blocks were cut into sections 6 μm thick and mounted on glass slides. Tissue sections were processed for histology and stained either with hematoxylin and eosin (H&E), toluidine blue, or immunostained for LEKTI, Matriptase, or KLK5. For IHC staining, antigen retrieval was performed by boiling samples in 0.01 M sodium citrate buffer, pH 6, for 12 min. After non-specific antigen blocking was performed, the sections incubated overnight at 4 °C with the primary antibodies followed by incubation with appropriate biotin-conjugated secondary antibodies and the Vectastain-ABC Elite kit (Vector Laboratories, Burlingame, CA, USA). Staining was developed by incubation with 3,3′-diaminobenzidine (25 mg/mL, Merck) and H_2_O_2_ and the sections were counterstained with hematoxylin. Non-immune anti-rabbit IgG was used as negative control (3 μg/mL, Catalog# 011-000-003, Jackson ImmunoResearch Laboratories Inc., West Grove, PA, USA) for LEKTI, Matriptase, and KLK5. Images were obtained on an Olympus VS120 slide scanner (Olympus Corporation, Tokyo, Japan). Quantification of the total epithelial area, the stained epithelial area of each sample and counts of metachromatically stained mast cells were performed using the Image J software [22]. Briefly, for TMA slides stained for LEKTI or Matriptase, ratios between the total epithelial and immunolabeled epithelial area (μm^2^) were calculated and plotted as a percentage (%); zero values were included where samples lacked LEKTI staining. For mice skin samples, the height of the epidermis (epithelial thickness) and mast cell count per 10^3^ µm^2^ were quantified.

### 2.9. Mouse Skin Dissociation

Mouse dorsal skin was extracted from euthanized healthy WT, K5-LEKTI^+/0^, K5-Matriptase^+/0^, and K5-LEKTI^+/0^/K5-Matriptase^+/0^ FVB/NJ mice. The dorsal skin was shaved and a depilatory cream was applied to remove remaining fur. The depilated skin was cleaned with PBS before extraction. An area of 15 mm × 35 mm of back skin was excised and cut into three pieces before being put in a 50 mL tube with 20 mL of HBSS and quickly washed twice by vortexing. The tissue was drained and placed into a 50 mL conical tube containing 10 mL of pre-dissociation buffer (RT HBSS with phenol red, without calcium or magnesium, 5 mM EDTA, and 10 mM HEPES), incubated for 30 min at 37 °C with agitation, and vortexed for 10 s before being strained through a 70 μm strainer. Tissue pieces were transferred to a Petri dish and finely cut into small fragments (2.2 mm × 2.2 mm) that were transferred to a new tube containing fresh pre-dissociation buffer and incubated again for further 30 min, vortexed vigorously for 10 s, strained and washed with 50 mL HBSS to remove excess EDTA. Collagenase digestion of skin was performed by incubating the strained tissue pieces in a new 2 mL Eppendorf tube containing 1.5 mL fresh digestion buffer (HBSS supplemented with 1 mg/mL collagenase D (Roche, Basel, Switzerland), 1 mg/mL collagenase type IV (Worthington Biochemical Co., Lakewood, NJ, USA), 100 μg/mL DNAse, 1 mM CaCl_2_) and 100 mg of Precellys^®^ Zirconium-Oxide Beads of 1.4 mm (Bertin Technologies). The tissue was incubated for 1–2 h at 37 °C with agitation and was briefly vortexed every 15 min. After dissociation, the contents were filtered through a 70 μm strainer into a new 50 mL conical tube, and flow-through containing the digested tissue was washed with 50 mL of DMEM with 10% FBS, centrifuged for 5 min at 350× *g*, and the cell pellet was resuspended in PBS for flow cytometry analysis.

### 2.10. Flow Cytometry

Skin isolated total cells were counted using an Automated Cell Counter (Countess I, Invitrogen, Thermo Fisher Scientific). The cell suspension (2.1–13 × 10^5^ cells/sample) was incubated for 15 min at RT with fixable dead cell stain (Thermo Fisher Scientific) and washed with AutoMACS Rinsing Solution containing 0.5% BSA (Mylteni Biotec, Bergisch Gladbach, Germany). The cell suspensions were then incubated for 10 min at 4 °C with anti-CD16/CD32 monoclonal antibody, followed by incubation for 30 min at 4 °C with anti-CD45, anti-Ly6G, anti-CD11c, anti-MHCII, and anti-CD64. The samples were acquired using a BD FACSCanto II cytometer and CellQuest software (BD Biosciences). Total events per sample were collected and analyzed according to size and granularity, single events, live cells, and fluorescence intensity using FlowJo software (BD Biosciences). The following gating strategy was used: leukocytes (CD45^+^) in total live and single cells; Ly6G^+^ (neutrophils) and Ly6G^−^ in CD45^+^ cells; dendritic cells (DC–CD11c^+^MHCII^High^) and myeloid cells (MY–CD11c^+^MHCII^+^) in total Ly6G^−^ cells, and macrophages (CD64^+^) in total myeloid cells.

### 2.11. Chemical Carcinogenesis

The shaved dorsal area of mice was treated with five applications of 25 μg of 7,12-dimethylbenzanthracene (DMBA) diluted in 100 μL of acetone. Applications started at five weeks of age and were performed every three weeks. The tumor incidence and size in the carcinogen-treated mice were monitored every three weeks. Mice with ulcerating tumors or tumors reaching a diameter of >2 cm were euthanized before the termination of the study at 48 weeks of age. Tumors and organs were collected and fixed and processed for histology.

### 2.12. PAR-2 Activation Assay

The PAR-2 activation assay was performed as previously described [10]. Briefly, HEK293T cells were cultured in DMEM and co-transfected with pCDNA3.1-PAR-2 (50 ng), pRL-Renilla luciferase (20 ng), and SRE-Firefly luciferase (50 ng) plasmids. At 16 h post-transfection, cells were treated for 6 h with 100 nM hrKLK5, or 15 nM hrMatriptase, which was used as a positive control. PAR-2 activation was measured using the Dual-Glo Luciferase Assay System according to the manufacturer’s instructions (Promega, Madison, WI, USA). Luminescence was measured using a Victor X3 plate reader (Perkin Elmer, Waltham, MA, USA), and SRE activation was determined as the ratio of firefly to Renilla luciferase counts.

### 2.13. TEAD Activation Assay

To measure TEAD activity, HEK293T cells in 12-well plates were co-transfected overnight with 8 × TEAD-Luc (0.25 μg/cm^2^) and pCDNA3.1-PAR-2 (50 ng). The next day, cells were serum-starved overnight and stimulated with 10 nM hrKLK5 for 6 h; luciferase activity was measured using a Dual-Glo Luciferase Assay Kit (Promega) and a Microtiter plate luminometer (SpectrMax iD3, Molecular Devices LLC, San Jose, CA, USA). Luciferase normalization was performed in every case by co-transfecting a Renilla Luciferase Vector (0.025 μg/cm^2^) (Promega).

### 2.14. Cytokine Release

WT or KLK5 KO Cal 27 cells were plated at 2 × 10^5^ cells per well in 24-well plates and cultivated for 16 h in DMEM containing 10% FBS. Cells were then starved for 2 h before stimulation with hrMatripase for 24 h. Supernatants from cell cultures were collected and measured by ELISA using the DuoSet ELISA kits (R&D Systems) according to manufacturer’s protocols for human IL-8/CXCL8 (Catalog# PD8000C), TNF-alpha (Catalog# DY210), and IL1-beta (Catalog# DBL50).

### 2.15. Scratch Assay

The Scratch assay was performed as previously described^21^. Briefly, WT or KLK5 KO Cal 27 cells (2 × 10^5^ cells) were cultured on glass coverslips, serum-starved for 16 h and treated or not with hrMatriptase. At time “0” a 10 μL plastic tip was used to scratch the cells in the middle of each coverslip and wound closure was evaluated for up to 48 h. The coverslips were imaged at 0, 24, and 48 h after scratching using an DMI4000 Leica epifluorescence microscope. The FIJI image processing package [22] was used to measure the area between the edges of the scratch in all time points. The following formula was used to calculate the wound closure area: Wound Closure Area = Area [T0] − Area [Tn].

### 2.16. Statistical Analysis

GraphPad Prism 8 (GraphPad Software, Inc., San Diego, CA, USA) was used for data analysis and preparation of graphs. Normality tests were performed. Data from experiments with two groups were analyzed using a *t*-test. Data from experiments with three or more groups were analyzed using one-way analysis of variance test (ANOVA) and post-test as indicated in the figure legends.

## 3. Results

### 3.1. LEKTI but Not Matriptase Is Differentially Expressed in Human OSCCs

**Figure 1:**
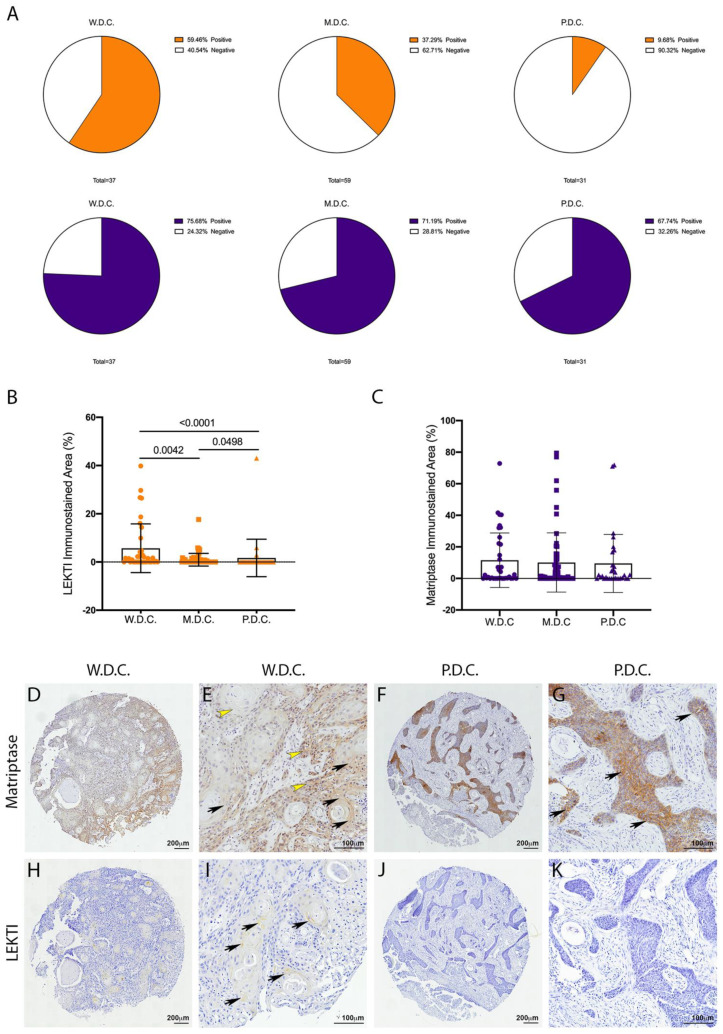
Unlike LEKTI, matriptase is not modulated in poorly differentiated carcinomas. (**A**) IHC staining for LEKTI (orange) and matriptase (purple) in human OSCCs TMAs (W.D.C. *n* = 37, M.D.C. *n* = 59, and P.D.C. *n* = 31) showed that the number of positive samples for LEKTI (top panel) prominently decreases in the less differentiate samples, while for matriptase (bottom panel) this number remains similar. (**B**) Quantification of stained area confirmed that LEKTI is significantly decreased in M.D.Cs. and P.D.Cs.; *p*-values (One-Way ANOVA) are displayed in the graph. (**C**) Quantification of stained area shows that matriptase expression does not vary among W.D.Cs., M.D.Cs., and P.D.Cs.; (**B**,**C**) Data are expressed in mean ± SD. (**D**–**G**) Representative images of matriptase IHC staining in well-differentiated and poorly differentiated carcinomas. (**H**–**K**) Representative images of LEKTI IHC staining in well-differentiated and poorly differentiated carcinomas. Black arrows show deeper staining, while yellow arrowheads show diffuse staining. Lower magnifications (**D**,**F**,**H**,**J**)-bar = 100 μm; Higher magnifications (**E**,**G**,**I**,**K**) bar = 200 μm; Counterstaining with hematoxylin to visualize tissue architecture.

Matriptase expression and activity are dysregulated in several human cancers [7]. In particular, matriptase was previously found to be ubiquitously expressed in HNSCCs of different anatomical locations and different presumed etiology [9]. Conversely, a recent study from our group showed that LEKTI was downregulated in OSCCs [21]. Because LEKTI has also been shown to inhibit a matriptase-driven proteolytic pathway in terminal epithelial differentiation [11], we hypothesized that it could play a role in matriptase mediated squamous cell carcinogenesis. To investigate this, we performed an immunohistochemical evaluation of TMAs comprised of human OSCCs of different anatomical locations of the oral cavity and of the following histopathological grades: well-differentiated carcinomas (W.D.C., *n* = 37), moderately differentiated carcinomas (M.D.C., *n* = 59), and poorly differentiated carcinomas (P.D.C., *n* = 31). While LEKTI expression is remarkably reduced in M.D.C.s and P.D.C.s, where the majority of the samples were negative (Figure 1A, top panel), matriptase expression was found in 70% of the samples in all three groups (Figure 1A, bottom panel). Quantification of the immunostained area in all samples confirmed decreased expression of LEKTI in less differentiated samples (Figure 1B), while matriptase expression remained unaltered among the different groups (Figure 1C). Matriptase expression was found throughout in both W.D.C. and P.D.C. samples (Figure 1D–G), and LEKTI expression was limited to well-differentiated cells in W.D.C.s (Figure 1I,J). These results are consistent with our previous work where increased protease/inhibitor ratio was associated with increased pathological grades and poor prognosis [21].

### 3.2. Generation of K5-LEKTI Mice

**Figure 2:**
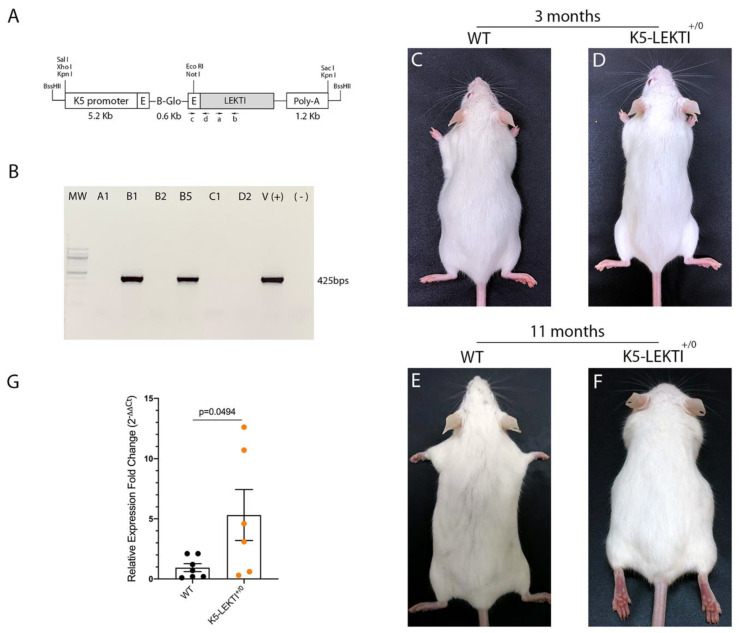
Generation of Keratin5-LEKTI transgenic mice. (**A**) Schematic structure of the K5-LEKTI transgene comprised of a bovine keratin-5 promoter (K5), rabbit-globin exons, a rabbit-globin intron, the mouse LEKTI cDNA and a rabbit-globin polyadenylation signal (PolyA). (a,b) position of primers used for qPCR and (c,d) genotyping. The linearized transgene vector was microinjected into the male pronucleus of FVB/NJ zygotes, which then were implanted into pseudopregnant mice. (**B**) LEKTI transgenic founders were genotyped by PCR using genomic DNA from tail biopsies with the primer pair indicated on the vector (c,d). Genotyping gel showing positive 425 bps amplified bands from founder mice. pBK5-LEKTI vector was used as template for positive control. (**C**–**F**) Images of 3 (**C**,**D**) and 11 months (**E**,**F**) old WT (**C**,**E**) and K5-LEKTI^+/0^ (**D**,**F**) mice showing no differences on the outward phenotype. (**G**) qPCR analysis of the epidermis of newborn WT (black dots) and K5-LEKTI^+/0^ (orange dots) mice show a five-fold increase of LEKTI mRNA expression in transgenic compared to WT mice. WT *n* = 7 and K5-LEKTI^+/0^ *n* = 6; values are expressed in mean ± SD. *p*-values (two-tailed unpaired parametric *t*-test) are displayed in the graph.

To further investigate the role of LEKTI in matriptase-dependent squamous cell carcinogenesis, we generated transgenic mice that express LEKTI in basal keratinocytes. Full-length murine LEKTI cDNA was cloned under the control of bovine keratin-5 promoter in the pBK5 vector (Figure 2A) [18]. Pronuclear injection of the transgene into multiple two-cell embryos generated five founders (Table 1). The keratin-5-LEKTI transgene was detected in two mice by PCR amplification of genomic tail DNA (Figure 2B). The transgenic founders were fertile and transmitted the transgene to the next generation. The mouse line FVB-K5-LEKTI-B1 was selected to establish the colony (K5-LEKTI^+/0^, Table 1). K5-LEKTI^+/0^ mice do not display any particular phenotype as evidenced by representative images of 3 and 11-month-old WT and K5-LEKTI^+/0^ mice (Figure 2C–F). qPCR analysis of RNA extracted from the epidermis of newborn WT and K5-LEKTI^+/0^ (Figure 2G) mice showed an average five-fold increase of LEKTI mRNA expression in the transgenic mice.

### 3.3. Co-Expression of LEKTI in Basal Keratinocytes Attenuates Matriptase-Dependent Premalignant Phenotype

**Figure 3:**
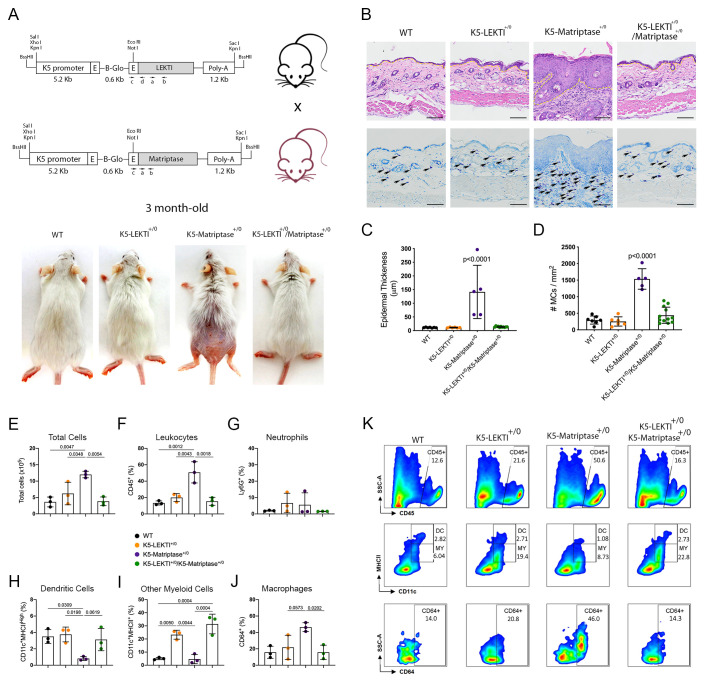
Co-expression of LEKTI attenuates matriptase-mediated premalignant skin phenotype. (**A**) The scheme shows the breeding of K5-LEKTI^+/0^ with K5-Matriptase^+/0^ mice and the resulting litter of WT, K5-LEKTI^+/0^, K5-Matriptase^+/0^, and K5-Matriptase^+/0^/K5-LEKTI^+/0^ mice. Images show the outward appearance of these mice at 3 months of age. Matriptase-induced alopecia and ichthyosis are considerably attenuated by co-expression of LEKTI in Matriptase^+/0^/K5-LEKTI^+/0^. LEKTI^+/0^ in 3-month-old mice. (**B**) Representative histological appearance of dorsal skin of littermate WT (first column), K5-LEKTI^+/0^ (second column), K5-Matriptase^+/0^ (third column), and K5-Matriptase^+/0^/K5-LEKTI^+/0^ mice (forth column) stained by H&E (top panels) and Toluidine Blue (bottom panels) at 3 months of age. Bars = 100 μm. Yellow dashed lines show the limits between epidermis and dermis. Black arrows show metachromatically stained dermal mast cells. (**C**) Quantification of epidermal thickness in littermate WT (*n* = 7, black dots), K5-LEKTI^+/0^ (*n* = 6, orange dots), K5-Matriptase^+/0^ (*n* = 5, purple dots), and K5-Matriptase^+/0^/K5-LEKTI^+/0^ (*n* = 11, green dots) at 3 months of age. Data are expressed in mean ± SD. (**D**) Quantification of the dermal mast cell accumulation in the skin of littermate WT (black dots), K5-LEKTI^+/0^ (orange dots), K5-Matriptase^+/0^ (purple dots), and K5-Matriptase^+/0^/K5-LEKTI^+/0^ (green dots) at 3 months of age. Data are expressed in mean ± SD. (**E**–**J**) Myeloid cellular infiltration was evaluated in skin samples by flow cytometry. (**E**) Total cells (×10^5^) were assessed by automated cell counter using trypan blue. The percentage of (**F**) leukocytes (CD45^+^), (**G**) neutrophils (Ly6G^+^ gated on CD45^+^), (**H**) dendritic cells (DC–CD11c^+^MHCII^High^ gated on CD45^+^Ly6G), (**I**) other myeloid cells (MY–CD11c^+^MHCII^+^ gated on CD45^+^Ly6G^−^), and (**J**) macrophages (CD64^+^ gated on myeloid cells) in the skin. WT (black dots), K5-LEKTI^+/0^ (orange dots), K5-Matriptase^+/0^ (purple dots), and K5-Matriptase^+/0^/K5-LEKTI^+/0^ (green dots). (**K**) Representative flow cytometry of skin samples by groups. Data are representative of one experiment (*n* = 3/group) and are expressed as means ± SD. *p*-values (One-Way ANOVA with Tukey’s post-hoc test) displayed in the graphs.

Matriptase induces malignant transformation when expressed in epithelial stem cells, and this process is preceded by a premalignant phenotype characterized by hyperplasia, dysplasia, and dermal inflammation [8]. To investigate whether LEKTI could play an inhibitory role in this context, we induced concomitant expression of both Matriptase and LEKTI in basal keratinocytes of transgenic mice. For that, transgenic mice expressing matriptase cDNA under the control of Keratin 5 promoter (K5-Matriptase^+/0^) were interbred with the K5-LEKTI^+/0^ mice (Figure 3A). Double-transgenic mice (K5-LEKTI^+/0^/K5-Matriptase^+/0^) showed a prominent rescue of matriptase-dependent premalignant phenotype. The animals were analyzed at 3 and 11 months of age, and co-expression of LEKTI and matriptase partially rescued matriptase-driven alopecia and ichthyosis (3-month-old mice, Figure 3A, and 11-month-old mice, Figure S1A). In addition, epidermal hyperplasia (Figure 3B and Figure S1B, top panels, yellow dashed line delimitates epidermal thickness) and dermal mast cell recruitment (Figure 3B and Figure S1B, bottom panels, black arrows) were also diminished, as verified by H&E histological evaluation and toluidine blue metachromatic staining, respectively.

Further quantification of epidermal thickness and dermal mast cell recruitment showed that matriptase-dependent premalignant phenotype is indeed ameliorated by concomitant LEKTI expression (Figure 3C,D and Figure S1C,D). To further investigate the role of LEKTI in the attenuation of matriptase induced dermal inflammation and myeloid cells infiltration into the skin, we performed flow cytometry analysis of cells isolated from digested dorsal skin samples of 3-month-old mice. In accordance with histopathological data, our results demonstrated that K5-Matriptase^+/0^ mice present a significant increase in the total number of inflammatory cells in the skin (Figure 3E). The inflammatory infiltration was confirmed by an elevated percentage of leukocytes (CD45^+^ cells) in K5-Matriptase^+/0^ mice, which decreased in K5-LEKTI^+/0^/K5-Matriptase^+/0^ mice (Figure 3F). Concerning myeloid cells, we analyzed neutrophils, dendritic cells, and macrophages. Although there was no difference in neutrophils (Ly6G^+^) among groups (Figure 3G), K5-Matriptase^+/0^ mice showed a significant reduction in the percentage of dendritic cells (D11c^+^MHCII^High^), not observed in the double-transgenic group (Figure 3H). Notably, a significant increase in the percentage of macrophages (CD64^+^) was observed in K5-Matriptase^+/0^ mice and rescued in double-transgenic mice (Figure 3J). Considering other myeloid cells (CD11c^+^MHCII^+^), LEKTI expression in both K5-LEKTI^+/0^ and K5-LEKTI^+/0^/K5-Matriptase^+/0^ mice induced a significant increase of this population in comparison to WT and K5-Matriptase^+/0^ mice (Figure 3I). Representative flow cytometry differences among groups are displayed in Figure 3K.

Overall, our data confirms that LEKTI can act as a tumor suppressor downstream of matriptase activation, as evidenced by its ability to modulate matriptase induced hyperplasia and inflammatory cell recruitment.

### 3.4. Co-Expression of LEKTI with Matriptase in Basal Keratinocytes Delays the Onset and Progression of Chemically Induced Carcinogenesis and Decreases KLK5 Expression

**Figure 4:**
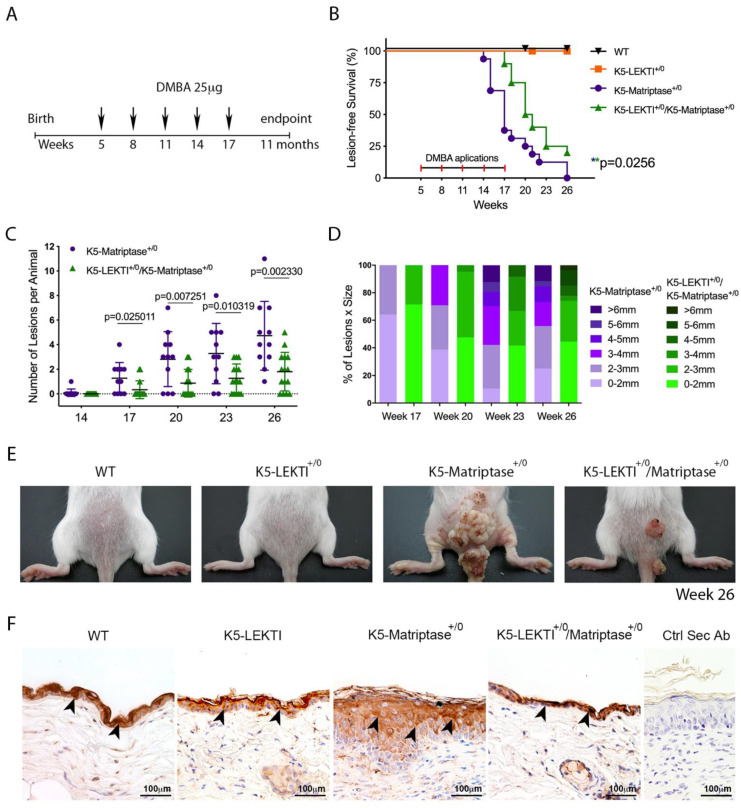
Co-expression of LEKTI with matriptase in basal keratinocytes delays the onset and progression of chemically induced carcinogenesis. (**A**) One-stage chemical carcinogenesis scheme: dorsal skin of mice was exposed 5 times to 25 μg of DMBA, starting at week 5 of age, every 3 weeks, and were followed for up to 48 weeks of age. WT (*n* = 20), K5-LEKTI^+/0^ (*n* = 17), K5-Matriptase^+/0^ (*n* = 11), and K5-Matriptase^+/0^/K5-LEKTI^+/0^ (*n* = 16). (**B**) Kaplan–Meier analysis of tumor-free survival. WT (black upside-down triangle), K5-LEKTI^+/0^ (orange squares), K5-Matriptase^+/0^ (purple dots), and K5-Matriptase^+/0^/K5-LEKTI^+/0^ (green triangles). (**C**,**D**) Matriptase induced tumor progression in K5-Matriptase^+/0^, and K5-Matriptase^+/0^/K5-LEKTI^+/0^ mice. Data are expressed in mean ± SD. (**C**) number of lesions and (**D**) percentage of lesion of each size in littermate K5-Matriptase^+/0^ (purple dots) and K5-Matriptase^+/0^/K5-LEKTI^+/0^ (green triangles) mice from 14 to 26 weeks of age. *p*-values (multiple *t*-tests) are displayed in the graphs. (E) Representative images of the outward appearance of littermate WT, K5-LEKTI^+/0^, K5-Matriptase^+/0^, and K5-Matriptase^+/0^/K5-LEKTI^+/0^ mice at 26 weeks of age. (**F**) Klk5 IHC staining of the skin of 3-month-old WT, K5-LEKTI^+/0^, K5-Matriptase^+/0^, and K5-Matriptase^+/0^/LEKTI^+/0^ mice. Black arrowheads indicate stained areas; Negative secondary antibody control; Bar = 100 μm.

Ras-dependent SCC is potentiated by the topical application of the genotoxic agent DMBA in K5-Matriptase^+/0^ mice [8]. To explore the inhibitory role of LEKTI in the initiation and progression of matriptase driven carcinogenesis, we used a one-stage carcinogenesis model where DMBA was applied to the dorsal skin of WT, K5-LEKTI^+/0^, K5-Matriptase^+/0^, and K5-Matriptase^+/0^/K5-LEKTI^+/0^ mice every three weeks for a total of five applications (Figure 4A). While WT and K5-LEKTI^+/0^ mice did not develop any lesions, all K5-Matriptase^+/0^ mice developed lesions at around 14–17 weeks of age. More importantly, there was an average 3-week delay in the emergence of lesions in K5-Matriptase^+/0^/K5-LEKTI^+/0^ mice compared to K5-Matriptase^+/0^ mice (Figure 4B). Tumor progression was also hampered by co-expression of LEKTI, as evidenced by a smaller number of lesions and the reduced average size compared to lesions of K5-Matriptase^+/0^ mice (Figure 4C,D). At 26 weeks of age, Matriptase^+/0^ mice display an increase in tumor size compared to the double-transgenic mice (Figure 4E). The observed delay in onset and progression of DMBA-induced carcinogenesis indicates that LEKTI could be working as a suppressor of squamous cell carcinogenesis in the context of dysregulated matriptase.

Since LEKTI is unable to inhibit matriptase directly [11], we hypothesized that there is another matriptase protease substrate in squamous cell carcinogenesis. In this respect, LEKTI inhibition of the matriptase-dependent proteolytic pathway could occur through the inhibition of epithelial kallikreins. In fact, immunohistochemical analysis of KLK5 expression in skin biopsies of 3-month-old mice showed that this protease is increased in K5-Matriptase^+/0^ mice. Notably, concomitant expression of LEKTI and matriptase in basal layer keratinocytes is sufficient to revert the aberrant KLK5 expression found in K5-Matriptase^+/0^ mice (Figure 4F).

### 3.5. KLK5 Activates YAP1-TAZ/TEAD Transcription via PAR-2 and Induces Matriptase-Mediated Release of IL-8 and Cell Migration in OSCC Cells

**Figure 5:**
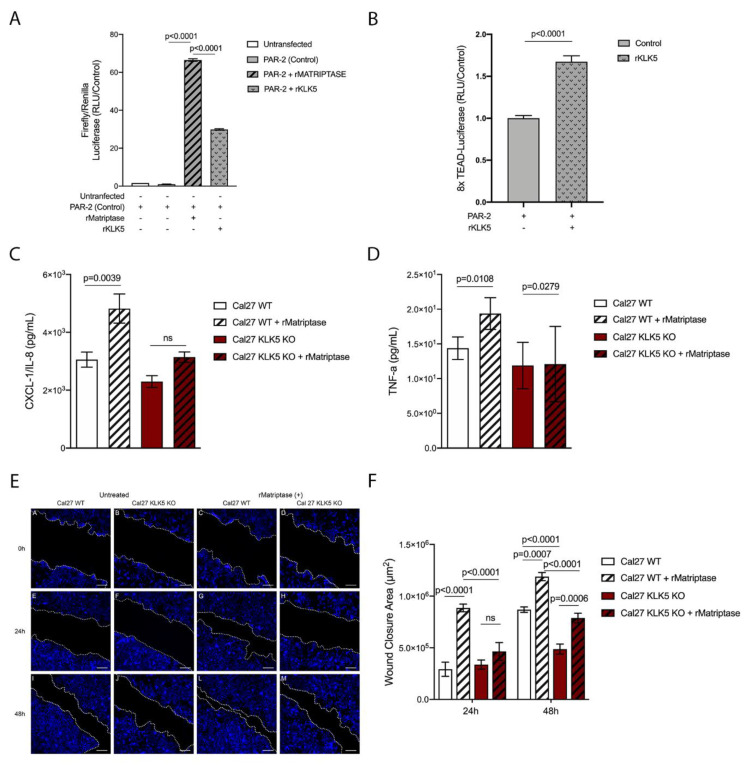
KLK5 activates YAP1-TAZ/TEAD transcription via PAR-2 and induces matriptase-mediated release of IL-8 and cell migration in OSCC cells. (A) KLK5-mediated PAR-2 activation analysis. HEK293T cells were transfected with pCDNA 3.1-PAR-2, pRL-Renilla luciferase and SRE-Firefly luciferase reporter plasmids and treated with hrKLK5 for 6 h, PAR-2 activation was measured by Luciferase activity. hrMatriptase was used as positive control for PAR-2 activation. *p*-values (One-Way ANOVA with Tukey’s post-hoc test) are displayed in the graphs. (**B**) Transcriptional activity of TEAD was also measured by luciferase assay with a reporter containing tandem TEAD-binding sites in HEK293T cells transfected with pCDNA 3.1-PAR-2 plasmid and treated with hrKLK5 for 6 h. *p*-values (two-tailed unpaired parametric *t*-test) are displayed in the graphs. (**C**,**D**) WT and KLK5 KO OSCC Cal 27 cell lines were serum-starved for 2 h, treated or not with hrMatriptase for 24 h, and proinflammatory cytokine release was evaluated by ELISA. (**C**) hrMatriptase treatment stimulated the release of CXCL-1/IL-8 in WT cells but not in KLK5 KO cells. (**D**) hrMatriptase treatment stimulated the release of TNF-α in both WT and KLK5 KO cells, although in KO cells this effect was very discreet. *p*-values (One-Way ANOVA with Tukey’s post-hoc test) are displayed in the graphs. (**E**,**F**) WT and KLK5 KO OSCC Cal 27 monolayers were serum-starved for 16 h, treated or not with hrMatriptase, scratched, and wound closure was evaluated for up to 48 h. (**E**) Representative images showing that hrMatriptase treatment induces wound closure in both WT and KLK5 KO cells (third and fourth columns) and this effect is partially inhibited in KLK5 KO cells (forth column) compared to WT cells (third column). Wound closure was also delayed in untreated KLK5 KO cells (second column) compared to WT cells (first column). (**F**) Quantification of the wound closure shows a delay in both matriptase treated (striped columns) and untreated (unstriped columns) KLK5 KO cells (red columns) when compared to WT cells (white columns) after 24 and 48 h. *p*-values (One-Way ANOVA with Tukey’s post-hoc test) are displayed in the graphs. (**A**–**F**) Data are representative of two independent experiments and values are expressed in mean ± SEM.

Because PAR-2 was previously shown to be essential for matriptase-driven premalignant progression and squamous cell carcinogenesis, we next investigated whether KLK5 activates PAR-2. For this purpose, we used a reconstituted cell-based assay in which HEK293 cells were transfected with a PAR-2 expression vector and a serum response element (SRE)-luciferase reporter plasmid. The transfected cells were then exposed to hrKLK5 to determine PAR-2 activation. As expected, KLK5 treatment was able to activate PAR-2 (Figure 5A, matriptase-dependent activation of PAR-2 was used as positive control). Importantly, KLK5-dependent PAR-2 activation was also responsible for an increase in Hippo-YAP1/TEAD transcriptional activity, as measured by luciferase assay with a reporter containing tandem TEAD-binding sites in HEK293T cells transfected with PAR-2 and treated with hrKLK5 (Figure 5B). Because PAR-2 activation also leads to NFkB activation, we sought to investigate the extent to which KLK5 contributes to the release of proinflammatory cytokines. To this aim, we used WT and KLK5 KO OSCC cell lines [21] treated or not with hrMatriptase for 24 h, and proinflammatory cytokine release was evaluated by ELISA. Matriptase-dependent release of IL-8 occurred only in cells expressing KLK5 (Figure 5C). TNF-α release was stimulated by matriptase in both WT and KLK5 KO cells (Figure 5D). Because both NFkB and Hippo signaling pathways may lead to increased cell migration in cancer [23,24], a scratch wound healing assay was used. WT and KLK5 KO OSCC Cal 27 monolayers were treated or not with hrMatriptase, scratched, and wound closure was evaluated for up to 48 h. Interestingly, matriptase-dependent wound closure after 24 h took place solely in cells expressing KLK5. After 48 h, however, hrMatriptase treatment induced wound closure in both WT and KLK5 KO cells (Figure 5E, third and fourth columns) and this effect was partially inhibited in KLK5 KO cells (Figure 5E, fourth column) compared to WT cells (Figure 5E, third column). Wound closure was also delayed in untreated KLK5 KO cells (Figure 5E, second column) compared to WT cells (Figure 5E, first column). Quantification of the wound closure confirmed a significant delay in both matriptase-treated (striped columns) and untreated (unstriped columns) KLK5 KO cells (red columns) when compared to WT cells (white columns) after 24 and 48 h (Figure 5F).

## 4. Discussion

This study aimed to explore the inhibitory role for LEKTI in matriptase-dependent SCCs and investigate additional players operating in this pathway. We showed here that LEKTI expression is either absent or remarkably reduced in less differentiated, more aggressive OSCCs, while matriptase expression is prevalent among OSCCs of different grades. Protease to inhibitor unbalance has been associated with uncontrolled proteolysis and malignant disorders before [25,26], and this result is in agreement with recent findings from our group, where LEKTI was shown to be downregulated in OSCCs in comparison with premalignant lesions and normal oral mucosa, with increased KLK5/SPINK5 mRNA (protease/inhibitor) ratio being associated with worse prognosis [21]. Furthermore, SPINK5 expression levels have been found to be downregulated in head and neck squamous cell carcinomas (HNSCCs) [27,28,29].

Consisting with an inhibitory role for LEKTI in the development of SCCs, we show that co-expression of LEKTI with matriptase in basal keratinocytes partially reverts matriptase-mediated premalignant phenotype, as evidenced by reduced alopecia, hyperplasia, and myeloid cell recruitment. In line with the fact that the tumorigenic microenvironment of SCCs often displays reduced quantities of myeloid CD11c^+^ dermal DCs, which indicates disruption in the DC immunostimulatory capacity [30,31], we showed a matriptase-dependent significant decrease in DCs. This was accompanied by increased macrophage infiltration, a phenotype rescued by co-expression of LEKTI in the skin of transgenic mice. Conspicuous influx of tumor-associated macrophages in SCC lesions can contribute to carcinogenesis and tumor growth [32]. More importantly, LEKTI expression also delayed the onset and progression of chemically induced carcinogenesis. Comparable inhibition was previously observed by co-expression of the matriptase cognate inhibitors hepatocyte growth factor inhibitors 1 and 2 (HAI-1 and HAI-2) [8,33].

In agreement with the fact that LEKTI inhibits matriptase-dependent skin desquamation in Netherton Syndrome by the direct inhibition of KLK5 [11] and our data shows that concomitant expression of LEKTI with matriptase rescues aberrant KLK5 expression. This indicates a role for KLK5 as a matriptase substrate. Dysregulated KLK5 has been implicated in a wide range of epithelial cancers [21,34,35,36,37,38]. Our data supports the existence of a matriptase-activated KLK5-dependent PAR-2 signaling axis in SCCs. PAR-2 is activated by several trypsin-like serine proteases, including matriptase and KLK5, signaling to various downstream pathways that modulate cell proliferation, migration and invasion, and cytokine production [10,21,36,39]. Regarding the potential mechanisms by which KLK5 can contribute to tumorigenesis, we show that KLK5 can induce PAR2 dependent activation of NFKB and Hippo-YAP1. Indeed, it has been shown that PAR-2 can lead to NFκB activation in inflammation and SCCs, and PAR-2 can be activated by KLK5 in OSCC [7,33,36,40,41]. In addition, PAR-2 activation by SLIGRL (PAR-2 peptide agonist) can result in YAP1 activation [42]. Hippo–YAP1/TEAD regulates organ size, tissue homeostasis, and tumorigenesis in mammals. Abnormal upregulation of this pathway occurs in many human malignancies and promotes tumor formation, progression, and metastasis [43,44].

Tumor invasion and metastasis are complex biological processes in which detachment and migration rely on matrix-degrading proteases. Knockdown of matriptase in endometrial cancer cells inhibits migration and invasion ability in vitro [45], and our recent work showed that CRISPR-mediated disruption of KLK5 blocks OSCC cell migration [21]. Our current results showed impaired matriptase-mediated migration in KLK5 KO OSCC cells, supporting a potential role of this pathway in cancer invasion and metastasis.

Taken together, our data support a model wherein matriptase activates PAR-2 via KLK5 and modulates SCC development and progression. This knowledge can contribute for the development of targeted therapeutics in HNSCC.

## 5. Conclusions

Our data identify a third signaling pathway for matriptase-dependent carcinogenesis in vivo, wherein matriptase activates PAR-2 via KLK5 and thus modulates SCC development and progression.

## Figures and Tables

**Table 1:** Generation of Keratin5-LEKTI transgenic mice.

Transgenic Founder	Gender ^a^	Skin Phenotype	LEKTI Expression
FVB-K5-LEKTI-A1	F	No	No
FVB-K5-LEKTI-B1	F	No	Yes ^b^
FVB-K5-LEKTI-B2	F	No	No
FVB-K5-LEKTI-B5	F	No	Yes
FVB-K5-LEKTI-C1	M	No	No
FVB-K5-LEKTI-D2	F	No	No

^a^—F: female; M: male. ^b^—Transgenic K5-LEKTI^+/0^ mice with higher expression of LEKTI.

## Data Availability

Data is contained within the article or supplementary material.

## Supplementary Materials

The following are available online at https://www.mdpi.com/article/10.3390/cancers13174395/s1, Figure S1: Co-expression of LEKTI attenuates matriptase-mediated premalignant skin phenotype.
