# Supplementary material for: Kallikrein 5 Inhibition by the Lympho-Epithelial Kazal-Type Related Inhibitor Hinders Matriptase-Dependent Carcinogenesis
Source: Cancers (Basel). 2021 Aug 31;13(17):4395. doi: 10.3390/cancers13174395 (PMC8431081; doi:10.3390/cancers13174395)
Supplement: Supplementary file 1 [file cancers-13-04395-s001.zip › cancers-1351020-supplementary.pdf]

Supplementay Information

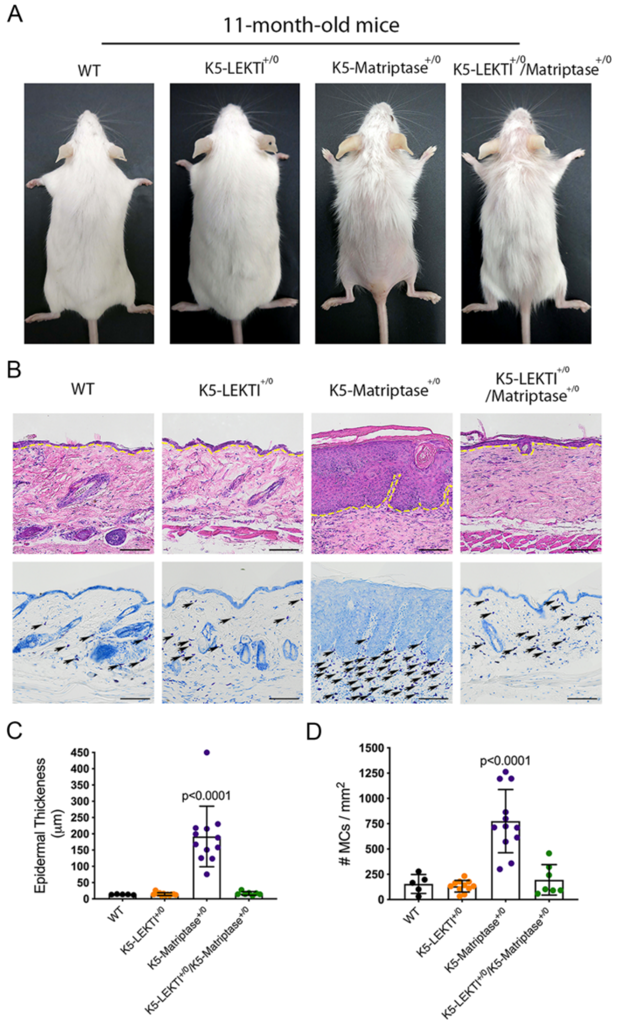

Supplementary Figure S1: Co-expression of LEKTI attenuates matriptase-mediated premalignant skin phenotype. A) The scheme shows the breeding of K5-LEKTI$^{+/0}$ with K5-Matriptase$^{+/0}$ mice and the resulting litter of WT, K5-LEKTI$^{+/0}$, K5-Matriptase$^{+/0}$, and K5-Matriptase$^{+/0}$/K5-LEKTI$^{+/0}$ mice. Images show the outward appearance of these mice at 11 months of age. Matriptase-induced alopecia and ichthyosis are considerably attenuated by co-expression of LEKTI in Matriptase$^{+/0}$/K5-LEKTI$^{+/0}$ in 11-month-old mice. **B)** Representative histological appearance of dorsal skin of littermate WT (first column), K5-LEKTI$^{+/0}$ (second column), K5-Matriptase$^{+/0}$ (third column), and K5-Matriptase$^{+/0}$/K5-LEKTI$^{+/0}$ mice (forth column) stained by H&E (top panels) and Toluidine Blue (bottom panels) at 11 months of age. Bars=100 µm. Yellow dashed lines show the limits between epidermis and dermis. Black arrows show metachromatically stained dermal mast cells. **C)** Quantification of epidermal thickness in littermate WT (n=7, black dots), K5-LEKTI$^{+/0}$ (n=6, orange dots), K5-Matriptase$^{+/0}$ (n=5, purple dots), and K5-Matriptase$^{+/0}$/K5-LEKTI$^{+/0}$ (n=11, green dots) at 11 months of age. *Data* are *expressed* in *mean* ± *SD*. **D)** Quantification of the dermal mast cell accumulation in the skin of littermate WT (black dots), K5-LEKTI$^{+/0}$ (orange dots), K5-Matriptase$^{+/0}$ (purple dots), and K5-Matriptase$^{+/0}$/K5-LEKTI$^{+/0}$ (green dots) at 11 months of age. *Data* are *expressed* in *mean* ± *SD*.
